# Characterizing ^13^C Spectral Assignments and Substituent Distributions of Hydroxypropylmethylcellulose Acetyl Succinate Using Dynamic Nuclear Polarization Nuclear Magnetic Resonance Spectroscopy

**DOI:** 10.1021/acs.molpharmaceut.5c00359

**Published:** 2025-08-29

**Authors:** Ronan P. Cosquer, Arthur C. Pinon, Mária Šoltésová, Lucy E. Hawarden, Anuji Abraham, Mike Tobyn, Frédéric Blanc

**Affiliations:** † Department of Chemistry, 4591 University of Liverpool, Liverpool L69 7ZD, U.K.; ‡ Swedish NMR Centre, Department of Chemistry and Molecular Biology, 3570 University of Gothenburg, Gothenburg 41390, Sweden; § Drug Product Development, 3970 Bristol-Myers Squibb, Moreton CH46 1QW, U.K.; ∥ Drug Product Development, Bristol-Myers Squibb, New Brunswick, New Jersey 08903, United States; ⊥ Stephenson Institute for Renewable Energy, University of Liverpool, Liverpool L69 7ZF, U.K.; # Leverhulme Research Centre for Functional Materials Design, Materials Innovation Factory, University of Liverpool, Liverpool L7 3NY, U.K.

**Keywords:** hydroxypropylmethylcellulose acetyl succinate, dynamic nuclear polarization, carbons connectivity, substituent distribution

## Abstract

Hydroxypropylmethylcellulose acetyl succinate (HPMC-AS) is the most widely used polymer in commercially available amorphous solid dispersions (ASDs), due to its ability to aid dissolution of poorly soluble drugs while impeding drug recrystallization. Nuclear magnetic resonance (NMR) spectroscopy is a well-suited approach to provide structural information on amorphous solids and access intermolecular interactions in multicomponent materials such as ASDs. The ^13^C spectral assignments for HPMC-AS differ in the literature, largely due to the significant structural complexity of this polymer, but are critical to identify drug-polymer interactions in ASDs containing HPMC-AS. A dynamic nuclear polarization (DNP) enhanced 2D ^13^C–^13^C refocused incredible natural abundance double quantum transfer experiment (INADEQUATE) spectrum is obtained to identify the one-bond ^13^C–^13^C connectivity in the polymer, which confirms the most recent ^13^C spectral assignments of HPMC-AS. Moreover, the spatial distribution of substituents in cellulose-based polymers is known to affect their physical properties and hence the dissolution or absorption of a formulated drug. Here, we use the definitive ^13^C spectral assignments of HPMC-AS and exploit the relayed-DNP of enhanced 1D cross-polarization (CP) spectra to determine that the HPMC-AS substituents are homogeneously distributed in three commercial grades of the polymer. Now, NMR experiments performed on ASDs containing HPMC-AS can more accurately correlate observed drug-polymer interactions to specific sites of the polymer. Therefore, a greater understanding into the mechanisms by which HPMC-AS stabilizes amorphous drugs.

## Graphical abstract

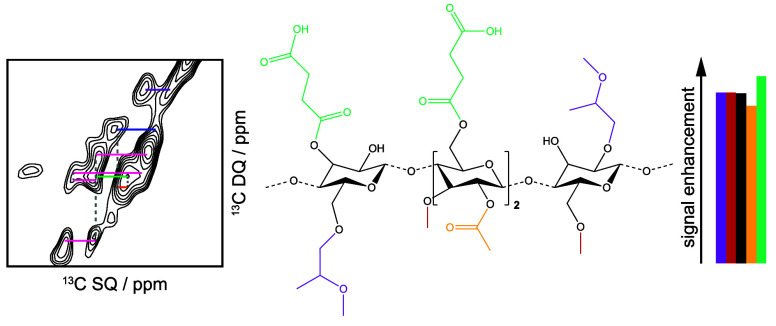

## Introduction

One of the greatest challenges faced in drug development is the poor bioavailability of active pharmaceutical ingredients (APIs),^1^ with approximately 90% of APIs in drug discovery pipelines exhibiting low aqueous solubility which inhibits their bioavailability and therefore, biological efficacy.^2^ Hydroxypropylmethylcellulose acetyl succinate (HPMC-AS) is used to aid dissolution of APIs in amorphous solid dispersions (ASDs) by stabilizing the more soluble amorphous state of the drug, dispersed in the polymer matrix (Figure 1).^3^ The opportunity to control drug-polymer miscibility and interactions that prevent drug recrystallization with different grades of HPMC-AS make it the most widely used polymer in commercially available ASDs.^4–6^

**Figure 1:**
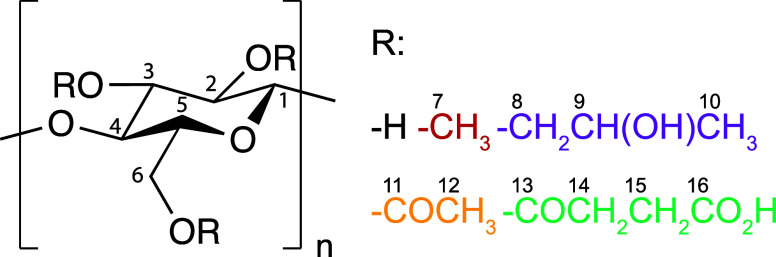
Structure of HPMC-AS and its different methyl (red), hydroxypropyl (purple), acetyl (yellow) and succinoyl (turquoise) substituents around the cellulose backbone (black). Chemically unique carbon environments are numbered for spectral assignments.

Solid-state nuclear magnetic resonance (NMR) spectroscopy is one of the most powerful approaches to gain noninvasive atomic level structural information for how HPMC-AS stabilizes amorphous drugs.^7^ Spectral assignments are critical to provide this understanding, in particular to identify intermolecular interactions within the ASD. Therefore, it is essential to accurately and fully assign the ^13^C magic angle spinning (MAS) NMR spectrum of HPMC-AS. However, this spectral assignment is challenged by the amorphous nature of HPMC-AS giving rise to inhomogeneous broadening yielding multiple overlapping ^13^C signals; the various degrees of substitution around the central cellulose background; the different ratios of acetyl and succinoyl groups, and, perhaps, by subtle changes in the HPMC-AS structures from various manufacturing processes.

The assignments of the ^13^C MAS NMR spectrum of HPMC-AS were first proposed using ^13^C cross-polarization (CP) and ^13^C-edited spectra (which are a well-established approach for differentiating carbon multiplicities) for HPMC-AS and selectively substituted hydroxypropylmethylcellulose (HPMC) polymers with acetyl (A) and succinoyl (S) groups only,^8^ with comparisons to known chemical shifts for the equivalent carbon environments of cellulose and HPMC.^9,10^ However, these assignments have recently been challenged based on expected increases in ^13^C chemical shift upon ether formation for selected carbon environments in HPMC-AS.^11^ This yields two different proposed assignments for carbons C2–C6 and C9 (see Figure 1 for carbon labels) of HPMC-AS, including different distinct chemical shifts for the ethers and esters of carbons C2, C3 and C6. Nearly quantitative peak integrals and empirical chemical shift predictions further supported and agreed with assignments from 2D solution state correlation experiments for similar cellulose-based polymers. Additionally, alternative ^13^C-edited NMR experiments were also used to facilitate spectral assignments.^12^ However, the effectiveness of these experiments is somehow limited in the presence of signal overlap or when there are multiple carbons of the same multiplicity, as observed for carbons C2–C5 of HPMC-AS. Therefore, the need for unambiguous ^13^C spectral assignment of HPMC-AS requires new, conclusive experimental data. One of such unequivocal approaches is to probe the carbon connectivities within the polymer network using the robust ^13^C–^13^C refocused incredible natural abundance double quantum transfer experiment (INADEQUATE). This experiment identifies the ^13^C–^13^C connectivities through their scalar ^1^ *J*_13C–13C_ coupling, as directly bonded ^13^C spins can be identified from their common double quantum frequencies.^13^

The 1.1% natural isotopic abundance of ^13^C implies that a ^13^C–^13^C spin pair has a probability of only 0.01%, therefore an additional approach is required to observe this spin pair which can be achieved from either ^13^C enrichment or dramatic increase in NMR sensitivity. HPMC-AS is a biomass derivative synthesized from HPMC which is produced from cellulosic tree pulp and thus ^13^C enrichment is largely unachievable, so another method is needed for the orders of magnitude increase in sensitivity required for ^13^C–^13^C refocused INADEQUATE acquisition. Dynamic nuclear polarization (DNP) offers such sensitivity gain by increasing the signal intensity using a transfer of the large polarization of unpaired electron spins to the nuclei of interest through microwave (μw) irradiation.^14–17^ Functionalized bisnitroxide biradicals are often used as an electron source for polarization transfer with the efficiency of polarization transfer increasing with increased electron spin relaxation times.^18^ Particles are impregnated with the radical solution, that is a nonsolvent to the sample of interest, then continuous microwave irradiation generates ^1^H (or other) nuclear hyperpolarisation. Polarization is transported from the surface to the core of the particles through ^1^H–^1^H spin diffusion then to the nuclei of interest through CP. DNP therefore presents new possibilities to obtain 2D correlation experiments at natural abundance for low sensitivity NMR-active nuclei such as ^13^C–^13^C refocused INADEQUATE^19,20^ and ^15^N–^13^C double CP-based heteronuclear correlation (DCP-HETCOR) experiments,^21,22^ which would simply not be accessible at natural abundance without the achieved signal enhancement. Moreover, the ^13^C–^13^C refocused INADEQUATE spectra of methylcellulose model compounds (a much structurally simpler biopolymer than HPMC-AS) (see Note Added in Proof for hydroxyethyl cellulose) have also been obtained with DNP as a generalizable method for spectral assignments of cellulose ethers.^23^ More generally, the sensitivity increase from DNP MAS NMR has been exploited in the pharmaceutical sciences for detection of dilute APIs in polymer excipients, identification of intermolecular interactions between an API and excipients and characterization of drug delivery systems.^24,25^

Another opportunity of DNP MAS NMR is for the understanding of the spatial distribution of polymer functional groups throughout the bulk polymer backbone, which has been demonstrated for different samples of HPMC with relayed-DNP.^26^ This approach relies on the gradient of polarization from the surface to the core at the steady state with the different DNP-enhancements of the functional groups of the polymer providing information on their distribution throughout the bulk. This is important as variations in spatial distributions of these functional groups could affect the absorption of a formulated drug in an ASD.^27^

Here, we confirm the most recent ^13^C spectral assignment of HPMC-AS via the acquisition of a DNP-enhanced 2D ^13^C–^13^C refocused INADEQUATE spectrum and determine that the substituents are homogeneously distributed in three different commercial grades of HPMC-AS from relayed-DNP.

## Experimental Section

All HPMC-AS samples were obtained from Shin-Etsu Chemical Co., Ltd. where they are synthesized from the addition of acetic anhydride and succinic anhydride to a solution of HPMC and glacial acetic acid in the presence of sodium acetate.^28^ The three polymer grades studied here were labeled as LF, MF and HF for low (L), medium (M) and high (H) acetyl/succinoyl ratios, respectively, with F denoting the fine particle size (5 μm). Dry powdered polymer samples were impregnated with 10–20 mM of the DNP polarizing agent AMUPOL^29^ in D_2_O/H_2_O of slightly different volume to volume ratio, as detailed in Table S1, and mixed together to produce a tacky-textured solid paste.^30^ A visual inspection of the sample confirmed homogeneous wetting. Crystals of KBr were added at the bottom of the rotors during the sample preparation to allow for temperature calibration from ^79^Br *T*_1_ measurements.^31^

DNP MAS NMR experiments were recorded on a Bruker AVANCE III HD NMR spectrometer with a 9.4 T wide-bore magnet and a 263.334 GHz gyrotron source, using a Bruker 3.2 mm low temperature double resonance DNP ^1^H/^13^C MAS NMR probe tuned to ^1^H at ν_0_(^1^H) = 400.1 MHz and ^13^C at ν_0_(^13^C) = 100.6 MHz. Samples were packed in 3.2 mm sapphire Al_2_O_3_ rotors, fit with Vespel caps and silicon plugs and spun under MAS at ν = 8 kHz. ^1^H pulses and SPINAL-64 ^32^ heteronuclear decoupling during all ^13^C detection were carried out with radiofrequency (rf) field amplitudes of 100 kHz. Hartmann–Hahn^33^ matched conditions for CP were achieved using a ^13^C rf amplitude of 69 kHz ramped from 70 to 100% for maximum signal at a ^1^H rf field amplitude of 100 kHz. Contact times for all CP-based experiments were 1 ms. To record the ^13^C–^13^C refocused INADEQUATE spectrum, a τ spin–echo evolution time of 2.5 ms was used for the (τ-π-τ) spin–echo.^34,35^ Spectra were recorded with recycle delays of 1.3 times the ^1^H spin–lattice relaxation times *T*_1_ (measured via a standard saturation recovery experiment). ^1^H–^13^C CP experiments using dipolar dephasing echo filter with ^13^C π-pulses at 70 kHz and a τ-delay of three rotor periods were used for selective excitation of quaternary and CH_3_ carbons.^36^

Room temperature ^13^C CP MAS NMR experiments were recorded on a Bruker AVANCE III HD NMR 9.4 T spectrometer with a 4 mm triple-resonance HXY MAS probe in double resonance mode tuned to ^1^H at ν_0_(^1^H) = 400.1 MHz and ^13^C at ν_0_(^13^C) = 100.6 MHz. Samples were packed in 4 mm ZrO_2_ rotors and spun under MAS at ν = 8 kHz. ^1^H pulses and SPINAL-64 ^32^ heteronuclear decoupling during ^13^C detection were carried out with rf field amplitudes of 70 kHz. Hartmann–Hahn^33^ matched conditions for CP with 1 ms contact time were achieved using a ^13^C rf amplitude of 64 kHz ramped from 70 to 100% for maximum signal at a ^1^H rf field amplitude of 70 kHz.

^13^C spectra on all NMR systems were externally referenced to the silicon plug at 1.8 ppm which is equivalent to the tertiary carbon resonance of adamantane at 29.45 ppm.^37^ All NMR data were processed using standard procedures in Topspin software. Deconvolution of experimental spectra was carried out in Topspin 4.4.1 using the solid line shape analysis routine.

Scanning electron microscopy (SEM) was performed on a Hitachi S4800. Powder samples were spread over an adhesive carbon tape stuck on an aluminum SEM stub. The after formulated sample was dried in air at 50 °C for 30 min to remove excess water. In order to reduce charging effects, the samples were coated with a thin layer of gold.

Standard differential scanning calorimetry (DSC) was performed on a TA Instruments DSC Discovery using a TA-Tzero aluminum pan loaded with around 5 mg of sample. Standard DSC analysis involved cooling the sample from 293 to 183 K with a ramp of 5 K min^–1^ then after an isotherm of 5 min a ramp of 5 K min^–1^ was used to heat the sample back up to 293 K.

## Results and Discussion

### DNP-Enhanced ^13^C–^13^C Refocused INADEQUATE Spectrum of HPMC-AS

As mentioned previously, the structural complexity of HPMC-AS has resulted in differences in ^13^C assignments,^8,11^ namely for carbons C2–C6 and C9 (Table 1). Differences mainly arise from assignment of carbons C2/C3/C6 to either one chemical shift or separate chemical shifts for the ethers or esters of these carbons, depending on the different substituents at these positions of the cellulose backbone. This has led to assignment of the resonance at 80 ppm to either C4 or the ethers of carbons C2/C3, carbons C5/C9 at a chemical shift of 60 or around 75 ppm, and the presence of the ester of carbon C6 at a chemical shift of around 60 ppm, independent from the chemical shift of the C6 ether at 70 ppm.

**Table 1:** Comparison of HPMC-AS ^13^C Chemical Shifts (ppm) and Spectral Assignments that Differ in the Literature^a^

^13^C environment	C2 ether	C2 ester	C3 ether	C3 ester	C4	C5	C6 ether	C6 ester	C9
Pugliese et al.^8^	75	/	75	/	84	60	71	/	60
Zheng et al.^11^	82–85	71–73	84–87	75–77	75–76	73–77	67–72	61–62	73–74

a Current work supports the recent assignments of Zheng et al. / represents ^13^C assignments that were not previously considered in the literature.

In order to obtain optimum signal enhancements for successful 2D ^13^C–^13^C INADEQUATE spectra at natural abundance, optimization of the sample formulation for DNP MAS NMR was required and used standard procedure^38^ (including 20 mM radical concentration) with the results summarized in Table S1. SEM images (Figure S1) before and after impregnation with the radical solution showed an agglomeration of polymer grains and that the HPMC-AS morphology is unchanged following the DNP sample formulation. The functionalized biradical AMUPOL was chosen as the source of unpaired electron spins for polarization transfer, given the most reliable signal enhancements obtained on similar cellulose derived polymers.^39^ A polarizing matrix of D_2_O/H_2_O 9:1 (v/v ratio) was selected to inhibit the dissolution of the polymer in the matrix with the deuterated solvent present to reduce the electron and ^1^H relaxation rates, increasing the DNP transfer efficiency. DNP NMR experiments are performed at low temperatures around 100 K to reduce the electron relaxation rates and allow for sufficient polarization transfer from the radical. Solvents such as dimethyl sulfoxide and glycerol are often used as cryoprotectants to stop aggregation of the polarizing agent upon cooling, however, the glassy amorphous state of HPMC-AS already acts as a natural cryoprotectant.^23^ Performing these experiments at low temperatures can lead to apparent differences in spectra compared to corresponding room temperature data (Figure 2). There are no differences in the line width of peaks upon changing temperature due to the amorphous nature of the polymer resulting in broad resonances from the disordered molecular arrangement. However, there are relatively small changes in chemical shifts (from −0.5 to −2.5 ppm) for the peaks of HPMC-AS upon cooling from 298 to 107 K. Standard DSC measurements from 293 to 183 K indicate the absence of any thermal event upon cooling in this temperature range, in agreement with the known glass transition temperature (*T*_g_ = 392 K) and melting point (*T*_m_ = 443 K) of HPMC-AS,^4^ which can be further expected down to the lower 107 K temperatures at which the DNP MAS NMR experiments are performed at (Figure S2). Moreover, the lack of a sharp peak at 4.8 ppm^40^ in the ^1^H spectrum of neat HPMC-AS highlights the absence of residual water that could impact the mobility and molecular state of the polymer during NMR measurements.

**Figure 2:**
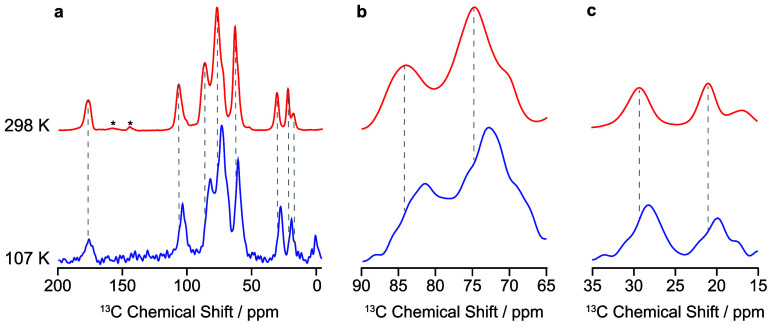
(a) Full width ^13^C CP MAS NMR spectrum of HPMC-AS (LF) acquired at 107 K (blue) and 298 K (red). (b) Insert for the 65–90 ppm region where there is a change of up to −2.5 ppm upon cooling. (c) Insert for the 15–35 ppm region where there is a change of up to −1.5 ppm upon cooling. Dashed lines help visualize shifts in peak positions upon cooling. Signal at 1.8 ppm arises from the silicon plug used to seal the rotor. Spinning sidebands are indicated with asterisks (*).

An overall sensitivity enhancement (Σ) of 36 was achieved which enabled a ^13^C–^13^C refocused INADEQUATE spectrum to be acquired for HPMC-AS (LF) in a few days (Figure 3). The experiment produces a 2D spectrum with the double quantum frequency in the indirect F1 dimension corresponding to the sum of the two single quantum frequencies of the two scalar *J* coupled carbons (and thus directly bonded) and correlates in the direct F2 dimension with the two corresponding ^13^C chemical shifts. Therefore, this data set enables the corresponding ^13^C–^13^C connectivities in the polymer network to be determined.

**Figure 3:**
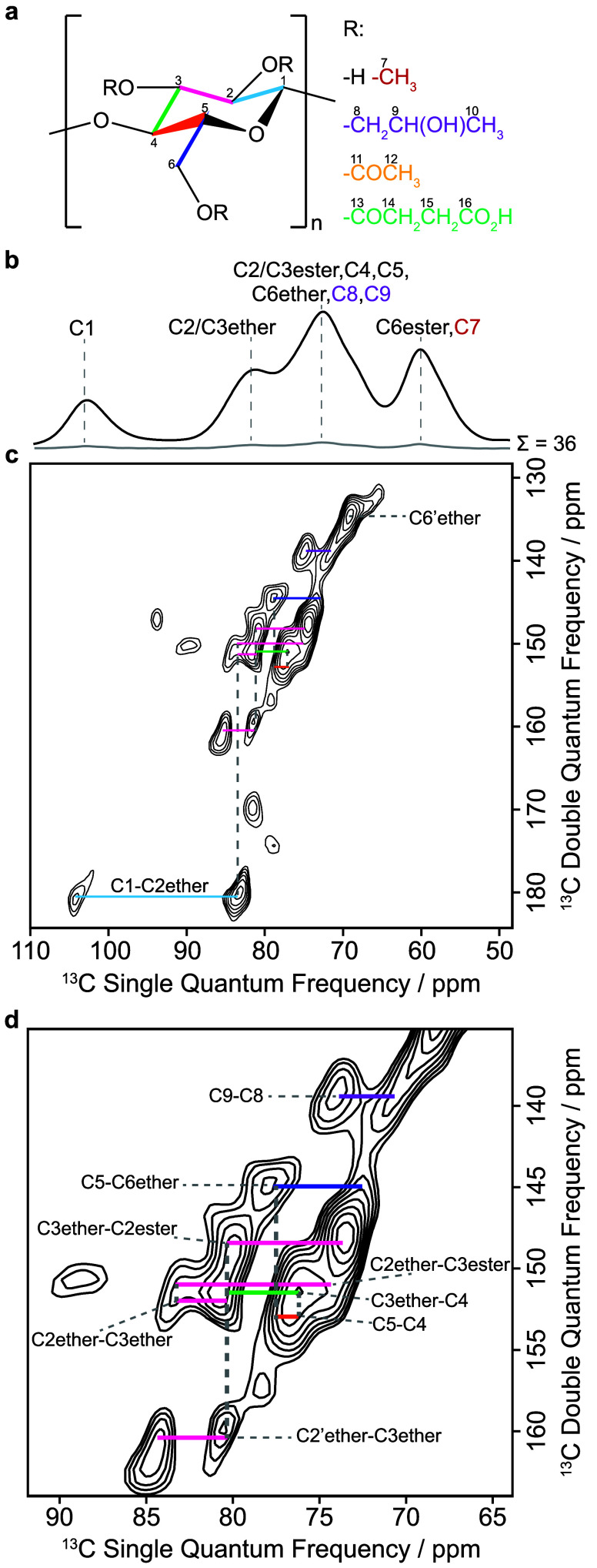
(a) Structure of HPMC-AS with numbered carbon atoms and color-coded bonds for spectral assignments. (b) 1D DNP MAS NMR ^13^C CP spectrum of HPMC-AS (LF) for μw off (gray) and μw on (black) with an absolute DNP enhancement (∑) of 36 and peaks labeled with their corresponding ^13^C assignments. (c) 2D DNP MAS NMR ^13^C–^13^C refocused INADEQUATE spectrum of HPMC-AS (LF) with labeled color-coded correlations based on (a). (d) Zoomed in region of the INADEQUATE spectrum to highlight some of the correlations. Only the regions containing challenged ^13^C assignments^8,10^ are shown for clarity.

As the epimeric carbon C1 of HPMC-AS (Figure 3a) has an undisputed chemical shift of 101 ppm (Figure 3b), similarly to its native carbon in HPMC^41^ and other cellulosic materials,^9,10^ it is a starting point to assign the rest of the carbons in the polymer network. C1 at 104 ppm has a correlation with the ether of C2 at 83 ppm to give a double quantum frequency signal at 187 ppm at the sum of their single quantum frequencies (Figure 3c, light blue line). This allows us to discriminate the two ^13^C assignments of HPMC-AS in the literature as C1 would only have a correlation with C2 at 75 ppm in the assignments of Pugliese et al.^8^ (Figure S3b, red circle).

The C2 ether also has a correlation with both the ester of C3 at 75 ppm and the ether of C3 at 81 ppm, the latter also correlating with the C2 ester at 74 ppm (Figure 3d, pink lines). Two correlations are present for the C2 ether–C3 ether (C2 ether and C2′ ether) which represent the two different ethers present in the polymer with methyl and hydroxypropyl substituents. This confirms the assignment of the peak at 81 ppm to the ethers of carbons C2/C3 and the peak at 75 ppm to the esters of carbons C2/C3. Additionally, the C3 ether at 81 ppm correlates with C4 at 76 ppm (Figure 3d, green line). These observations are expected for the increase in ^13^C chemical shift of carbons C2 and C3 following ether formation, which leads to C4 appearing at a lower chemical shift of 76 ppm compared to pulp cellulose.^10^ The remaining observed correlations for C4, C5, C6 ether and C9 further agree with the more recently proposed ^13^C assignments of HPMC-AS (Figure 3c, orange, navy and purple lines).

Nearly quantitative multiCP spectra for HPMC-AS in the literature show the presence of two carbon environments at 60 ppm.^11^ As C5 and C9 are assigned at 78 and 74 ppm respectively, this leaves the remaining environment at 60 ppm to be the ester of C6. This completes the disputed ^13^C assignments of C2–C6 and C9 for HPMC-AS which are in agreement with those proposed by Zheng et al.^11^ (Figure 4 and Table S2). The remaining 50 ppm signal is left unassigned as previously observed for other methylcellulose compounds.^23^ Identification of directly bonded ^13^C spins through the INADEQUATE experiment has thus allowed us to provide definitive spectral assignments for HPMC-AS. With these available, the ^1^H assignments can be reported using ^1^H–^13^C HETCOR spectra and ^1^H MAS NMR spectra at fast MAS (60 kHz) for HPMC-AS available^42^ by identifying the one bond ^1^H–^13^C correlations (Table S3). Therefore, future ^1^H and ^13^C MAS NMR experiments on ASDs containing HPMC-AS can thus infer more accurate information on site-specific drug-polymer interactions that could explain the efficiency of this polymer.

**Figure 4:**
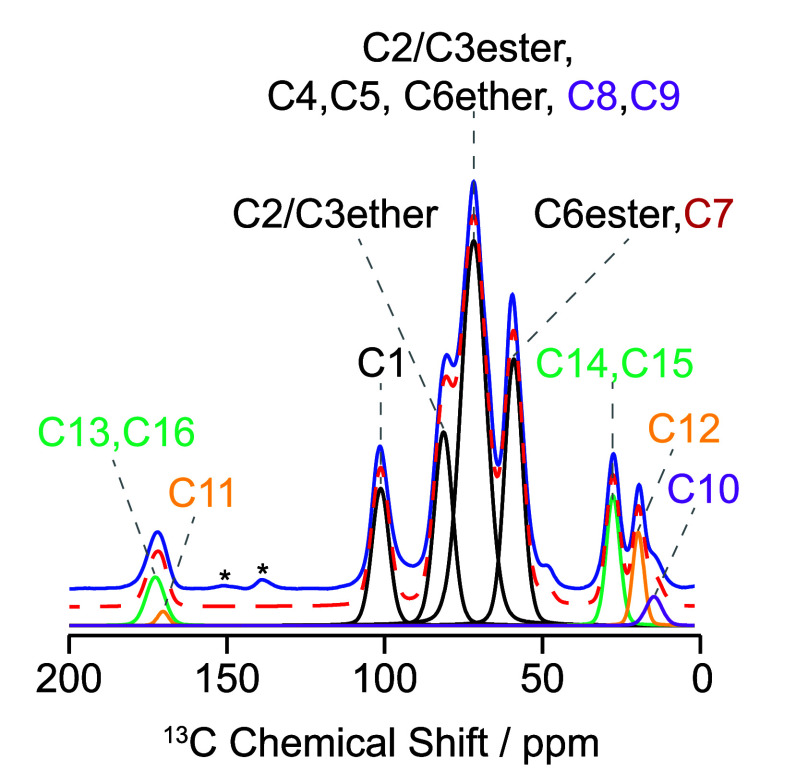
Full width DNP MAS NMR ^13^C CP spectrum of HPMC-AS (LF) (blue) and deconvoluted spectrum (red-dashed line) with color-coded assignments for the different carbon environments.

### Probing Substituent Homogeneity for Different Commercial HPMC-AS Grades

To efficiently stabilize an amorphous drug in a formulated ASD, HPMC-AS substituents that are homogeneously distributed across the cellulose backbone are preferable.^27^ The spatial distribution of polymer substituents (Figure 1) in three different grades of HPMC-AS were thus probed using relayed-DNP.^24^ Here, hyperpolarisation is transferred from the AMUPOL (situated in a frozen layer of the polarizing matrix at the surface of the HPMC-AS particles) to the core of the particles through ^1^H–^1^H spin diffusion. This leads to a polarization gradient from the surface to the core of the polymer particles that helps differentiate the spatial distribution of polymer substituents based on their NMR sensitivity gain compared to the homogeneously distributed cellulose backbone. Therefore, if a certain substituent is inhomogeneously concentrated more at the surface or core of the particles it will respectively have a greater or weaker gain in sensitivity compared to the inherently homogeneously distributed cellulose backbone. For each sample, experiments were obtained for the ^1^H–^13^C CP spectrum with and without microwave over three repeated measurements (Figure 5). DNP-enhancements were measured through the polarization transfer from the hyperpolarised ^1^H spins during the ^13^C CP experiment. Although DNP NMR experiments are typically conducted around 100 K, μw-induced sample heating can cause differences in ^1^H *T*_1ρ_ and hence different CP dynamics between μw on and μw off measurements. This could artificially enhance or reduce the signal intensity for some of the more mobile groups of the polymer which would lead to incorrect data interpretation.^26^ Above 130 K, these temperature fluctuations are easier to control and CP dynamics are less affected by microwave induced sample heating so all relayed-DNP data were collected at this 130 K controlled temperature by adjusting the experimental set up.^43^

**Figure 5:**
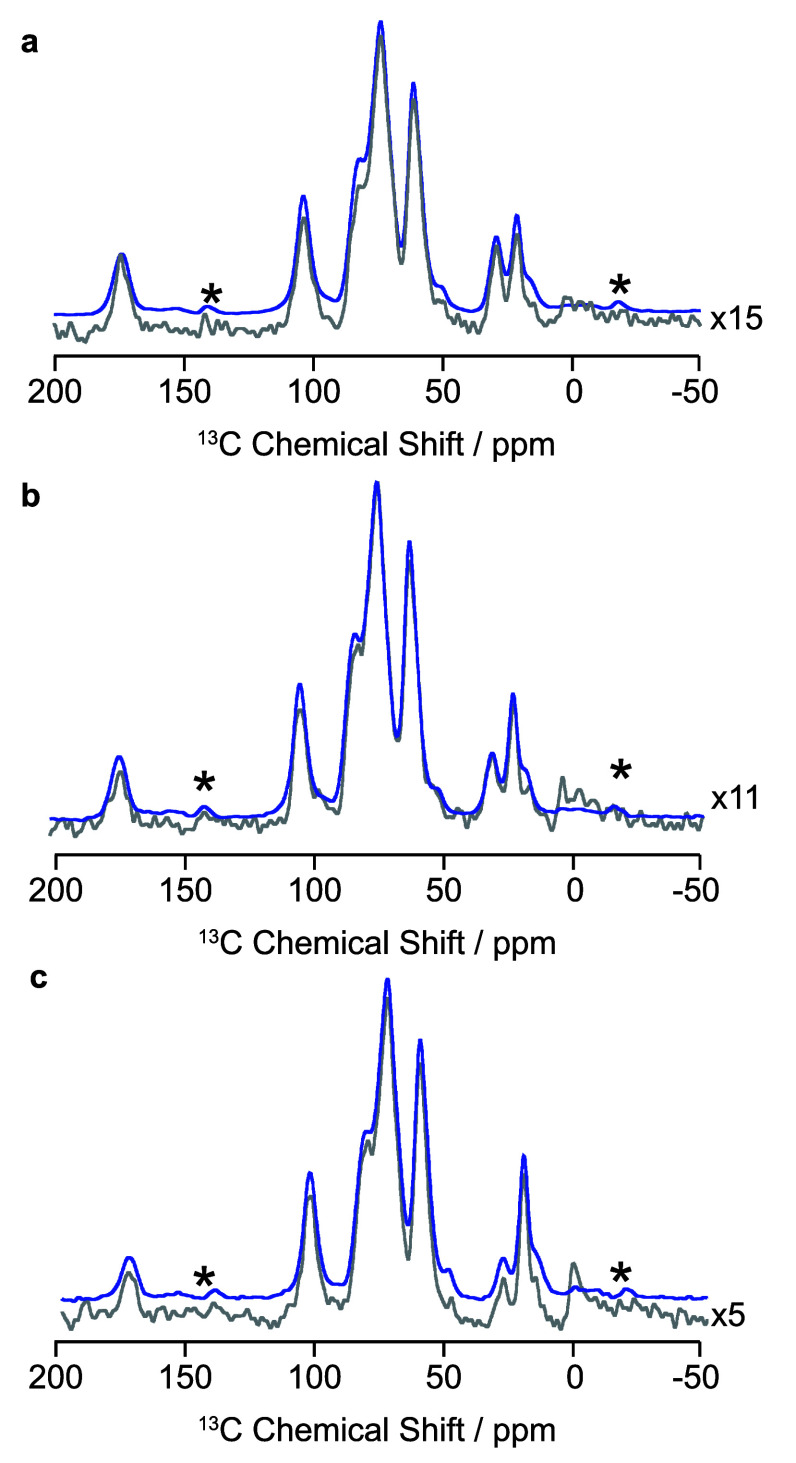
DNP MAS NMR ^13^C CP spectra with μw off (gray) and μw on (blue) for (a) HPMC-AS (LF), (b) HPMC-AS (MF) and (c) HPMC-AS (HF). The μw off spectra are scaled for clear comparisons with the μw on spectra. Signal at 1.8 ppm arises from the silicon plug used to seal the rotor. Spinning sidebands are indicated with asterisks (*).

Figure 5 shows the ^13^C CP spectra for the three grades of HPMC-AS. Comparison of the enhancement for the cellulose backbone (105 ppm) with the enhancements for the substituents on the cellulose ring (hydroxypropyl at 17 ppm, methyl at 62 ppm, acetyl at 20 ppm and succinoyl at 30 ppm) reveals similar sensitivity gain for each peak. As the peak at 60 ppm has overlapping contributions from carbons C6 ester and C7, dipolar dephasing echo filters were used to selectively observe the quaternary and ^13^CH_3_ carbon signals (Figure S4).^26^ This further shows that the relative enhancement for the methyl substituent is the same as those for the acetyl/succinate substituents and hence the bulk cellulose backbone. Given the gradient of polarization transfer from the surface to the core of HPMC-AS particles, the experimental observation highlights that all the substituents are homogeneously distributed throughout the bulk polymer.

The enhancements for the different groups of HPMC-AS are summarized in Figure 6 for the three different grades of HPMC-AS (LF, MF and HF) over three repeated measurements (1, 2 and 3). Here, for each separate repeated measurement of the three grades of HPMC-AS, the enhancements for all the substituents are equal to that of the cellulose backbone. Therefore, this indicates a homogeneous distribution of all the substituents throughout the three polymers. Variation in the magnitude of the enhancement across the three repeated measurements (Figure 6b,c) is similar to that observed for HPMC and 3-*O*-methylcellulose,^26^ though importantly the equivalent enhancements of the different polymer’s substituents with respect to the cellulose backbone is repeatable. The differences in enhancement across the repeated measurements are an outcome of the sample formulation for DNP MAS NMR measurements.^24^

**Figure 6:**
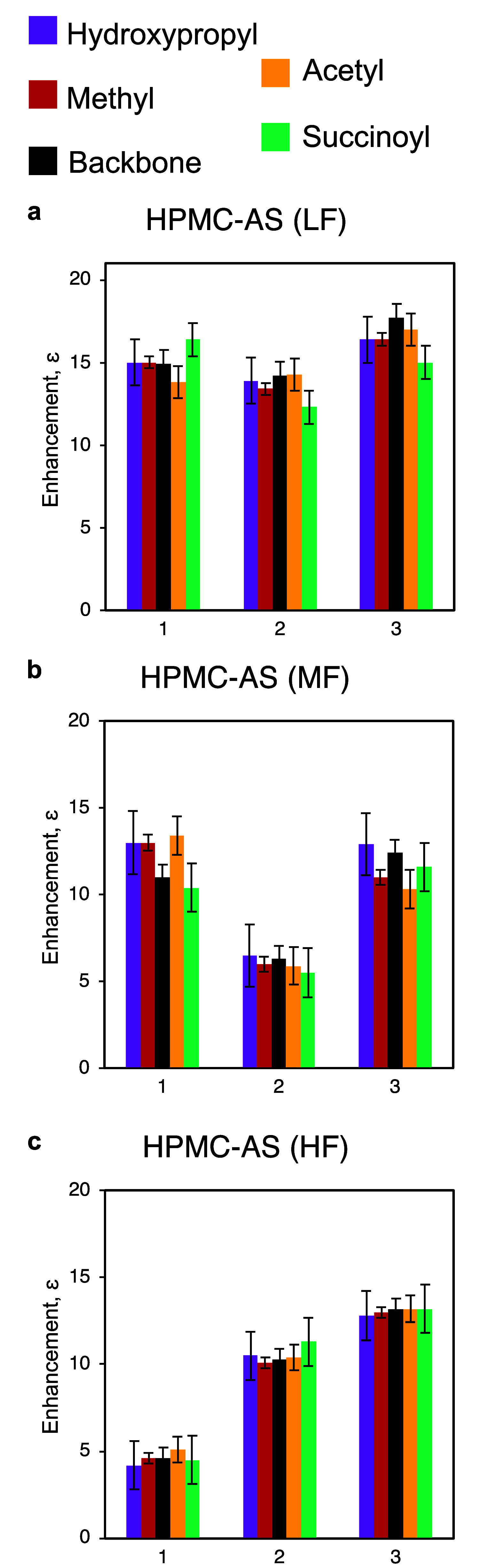
^1^H DNP enhancements measured through ^1^H–^13^C CP for (a) HPMC-AS (LF), (b) HPMC-AS (MF), (c) HPMC-AS (HF) over three repeats. The different components of HPMC-AS are color-coded as follows: hydroxypropyl (purple), methyl (red), cellulose backbone (black), acetyl (yellow) and succinoyl (turquoise). Errors in enhancement represent 1× standard error, with the calculation used detailed in the Supporting Information.

The homogeneity observed here is consistent with the synthetic pathway of HPMC-AS with the homogeneous addition of the acetyl and succinoyl substituents being done via esterification of HPMC in solution. Similar relayed-DNP experiments previously performed on different batches of HPMC (specifically selected to have identical chemical properties but significantly different physical properties) have identified differences in the distribution of polymer substituents as a consequence of the heterogeneous reaction conditions used to produce HPMC from pulp cellulose.^26^ One batch of HPMC had lower enhancements for the hydroxypropyl group of the polymer which indicated that this substituent was concentrated more in the core of the HPMC particles. Another batch of HPMC had equivalent DNP enhancements for all the polymer substituents which highlighted a homogeneous distribution. This highlights the unsurprising differences in HPMC substituent distribution from the heterogeneous manufacturing processes. Therefore, the three grades of HPMC-AS studied here are synthesized from a highly purified HPMC starting material with an already homogeneous distribution of methyl and hydroxypropyl substituents. It is known that in ASDs of acetaminophen and HPMC-AS, hydrogen bonding between the drug amide and HPMC-AS methoxy substituent stabilizes the amorphous drug,^42^ which illustrates the necessity for a homogeneous distribution of this substituent along the cellulose backbone of HPMC-AS. Hence, this characterization of the spatial distribution of polymer substituents along the HPMC-AS particle cross sections provides new insight into the efficient stabilization of a formulated drug with this polymer.

## Conclusions

A DNP-enhanced 2D ^13^C–^13^C refocused INADEQUATE spectrum was obtained for HPMC-AS (LF) which has enabled determination of definitive ^13^C spectral assignments for the polymer. These are in agreement with the more recently proposed assignments in the literature^11^ and confirm the presence of multiple ^13^C resonances for the ether/esters of the carbons on the polymer backbone, depending on the nature of the substituents. This approach highlights the necessity for definitive NMR experiments in obtaining spectral assignments for materials exhibiting many broad overlapping signals in their spectra. Now, ^13^C NMR experiments performed on HPMC-AS can more accurately relate findings to specific carbon sites of the polymer.

The homogeneity of HPMC-AS substituents in three different polymer grades was probed using relayed-DNP, which showed a homogeneous distribution throughout the polymer particles. Characterization of this homogeneity across the polymer cross section confirms the solution-based synthesis of HPMC-AS and provides insight into how this physical property of the polymer could impact its efficiency in commercial applications.

## Supplementary Material



## Data Availability

Data supporting the findings of this work are available from the University of Liverpool Research Data Catalogue, DataCat at https://doi.org/10.17638/datacat.liverpool.ac.uk/3037.

## Notes

Sample formulation (Table S1), ^13^C (Table S2) and ^1^H (Table S3) isotropic chemical shifts, SEM image (Figure S1), DSC (Figure S2), full width INADEQUATE spectra (Figure S3), ^13^C dipolar dephasing spectra (Figure S4) (PDF)

## Notes

Recently, ^13^C–^13^C DNP-enhanced refocused INADEQUATE experiments and room-temperature quantitative single-pulse ^13^C spectra have proven reliable tools to characterize the consistency and quality of hydroxyethyl cellulose in solid state formulations.^44^
