# Supplementary material for: Characterizing 13C Spectral Assignments and Substituent Distributions of Hydroxypropylmethylcellulose Acetyl Succinate Using Dynamic Nuclear Polarization Nuclear Magnetic Resonance Spectroscopy
Source: Mol Pharm. 2025 Aug 29;22(10):5870–8. doi: 10.1021/acs.molpharmaceut.5c00359 (PMC12505264; doi:10.1021/acs.molpharmaceut.5c00359)
Supplement: Supplementary file 1 [file mp5c00359_si_001.pdf]

Supporting Information

Characterising ^{13}C Spectral Assignments and Substituent Distributions of Hydroxypropylmethylcellulose Acetyl Succinate Using Dynamic Nuclear Polarisation Nuclear Magnetic Resonance Spectroscopy

Ronan P. Cosquer,[a] Arthur C. Pinon,[b] Mária Šoltésová,[b] Lucy E. Hawarden,[c] Anuji Abraham,[d] Mike Tobyn,[c] and Frédéric Blanc.[,a,e,f]*

[a]Department of Chemistry, University of Liverpool, Liverpool L69 7ZD, U.K., [b]Swedish NMR Centre, Department of Chemistry and Molecular Biology, University of Gothenburg, 41390 Gothenburg, Sweden, [c]Drug Product Development, Bristol-Myers Squibb, Moreton CH46 1QW, U.K., [d]Drug Product Development, Bristol-Myers Squibb, New Brunswick, New Jersey 08903, United States, [e]Stephenson Institute for Renewable Energy, University of Liverpool, Liverpool L69 7ZF, U.K., [f]Leverhulme Research Centre for Functional Materials Design, Materials Innovation Factory, University of Liverpool, Liverpool L7 3NY, U.K..

*Corresponding Author: frederic.blanc@liverpool.ac.uk

The overall sensitivity enhancement (Σ) is obtained from the following equation:

$$\Sigma = \varepsilon\theta\sqrt{\frac{T_1}{T_\mathrm{B}}}$$

where Ɛ is the ratio of the peak integrals with and without the microwave (normalised per mass of sample and number of scans), θ is the bleaching factor which is the ratio of the peak integrals with and without the radicals, T_1 is the ^{1}H T_1 without radicals and T_B is the ^{1}H DNP build up time measured through ^{13}C detected ^{1}H saturation recovery experiment.[1]

Table S1: Summary of the different sample preparations used for DNP MAS NMR experiments and the corresponding overall sensitivity enhancements. Sample **H** was used to calculate the bleaching factors (with T_1 = 1.4 s) as no radical was present.

Sample	AMUPOL (mM)	DNP solution	Mass of HPMC-AS (mg)	Volume of DNP solution (μL)	Ɛ	θ	T_B (s)[a]	Σ
**A**	20	1:9 (H_2O:D_2O)	41.2	164.8	26	0.9	0.6	36
**B**	10	1:9 (H_2O:D_2O)	17.3	17.3	13	0.5	1.0	8
**C**	10	1:9 (H_2O:D_2O)	20.6	41.2	14	0.5	0.8	9
**D**	10	0.2:99.8 (H_2O:D_2O)	23.9	23.9	7	0.5	1.0	4
**E**	10	2:8 (H_2O:D_2O)	15.2	15.2	8	0.5	0.9	5
**F**	20	1:9 (H_2O:D_2O)	15.1	30.2	12	0.5	0.7	8
**G**	20	1:9 (H_2O:D_2O)	23.3	186.4	22	0.8	0.4	34
**H**	0	1:9 (H_2O:D_2O)	30.3	121.2	1	1.0	1.4[b]	N/A
**I**	20	1:9 (H_2O:D_2O)	37.6	37.6	9	0.4	0.8	5
**J**	10	1:9 (H_2O:D_2O)	24.0	96.0	17	0.8	0.8	20

[a] Errors in the measured 1H T_1 and T_B values are ± 0.1 s. [b] T_1 value.

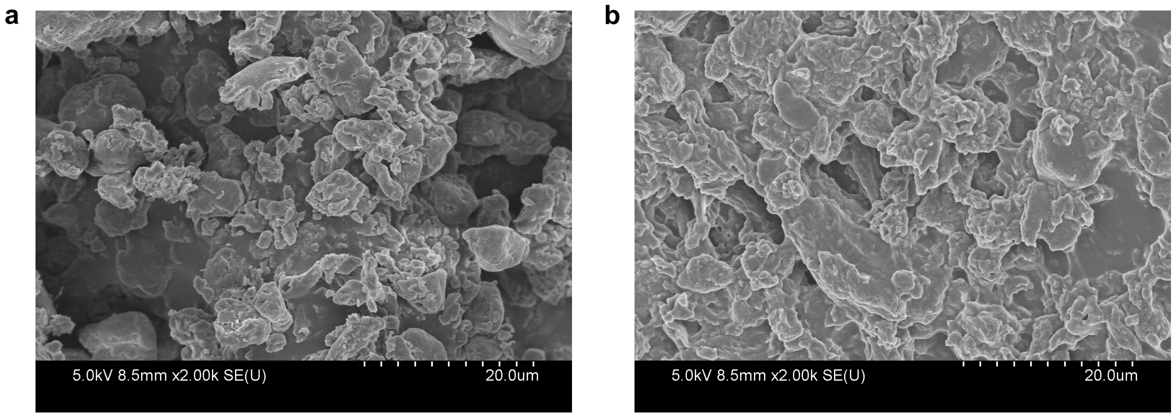

Figure S1: SEM images of HPMC-AS (LF) **a** before and **b** after impregnation with the DNP polarising solution.

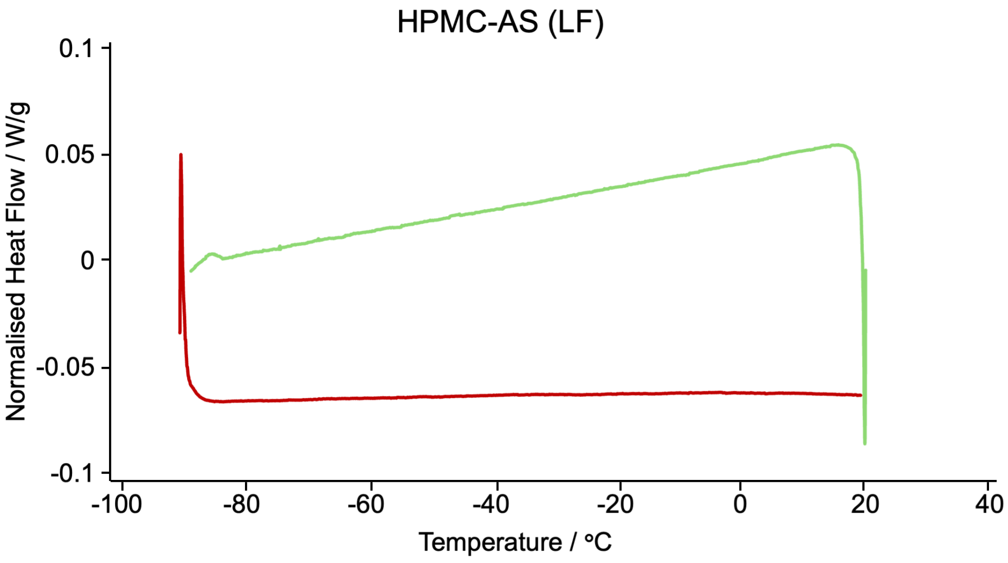

Figure S2: Standard DSC of HPMC-AS (LF) with cooling from 293 K to 183 K (green line), followed by heating back up to 293 K after a 5 minute isotherm (red line).

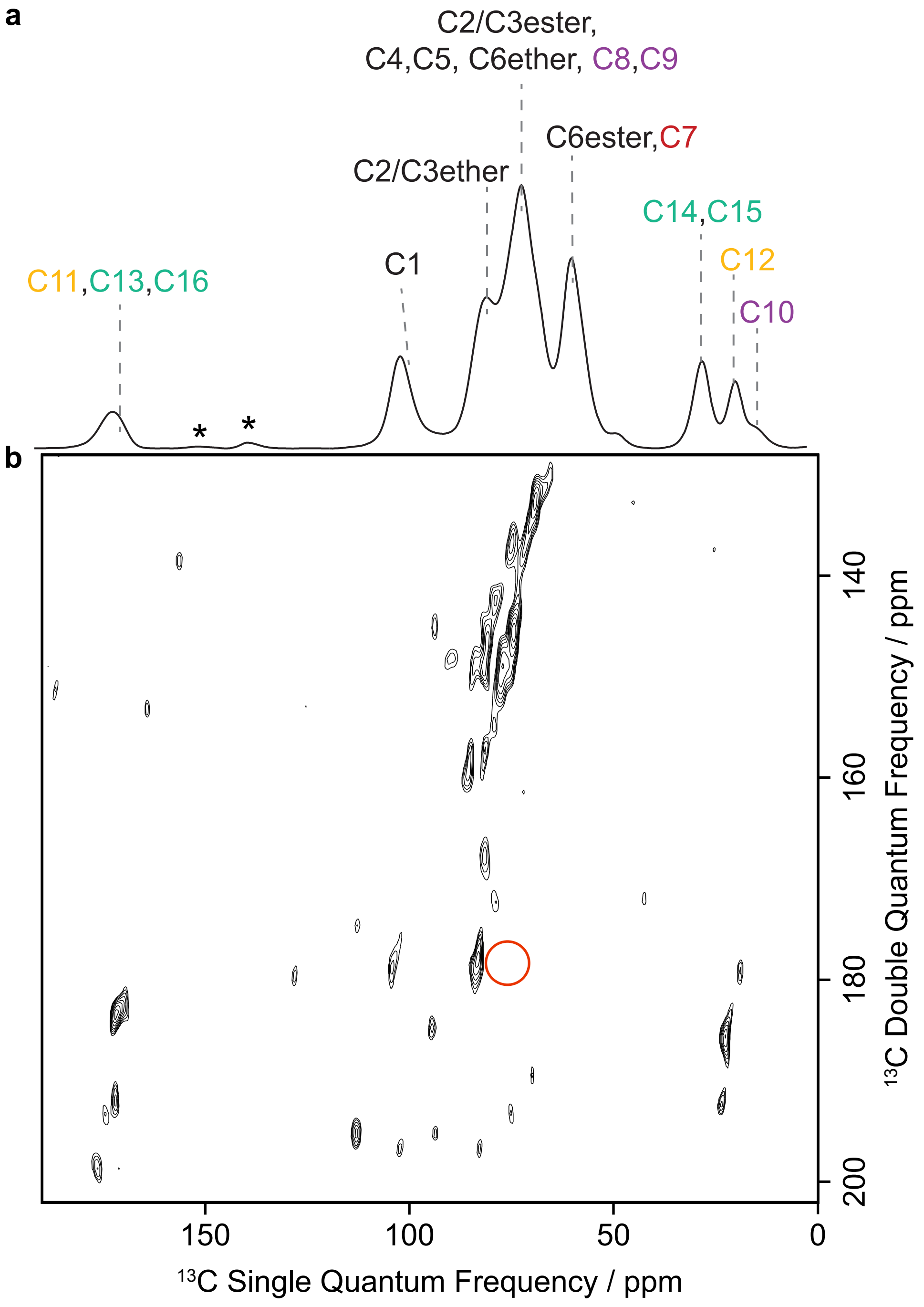

Figure S3: **a** Full width DNP MAS NMR ^{13}C CP spectrum of HPMC-AS (LF) with colour-coded assignments for the different carbon environments. **b** Full width 2D DNP MAS NMR ^{13}C-^{13}C refocused INADEQUATE spectrum of HPMC-AS (LF). The red circle highlights where the C1-C2 correlation would be for the erroneous assignment of HPMC-AS.[2] Spinning sidebands are indicated with asterisks (*).

Table S2: Comparisons of the previously proposed HPMC-AS ^{13}C chemical shifts (ppm) in the literature with those obtained here. / represents ^{13}C assignments that were not previously considered in the literature.

^{13}C environment	Temperature	C1	C2 ether	C2 ester	C3 ether	C3 ester	C4	C5	C6 ether	C6 ester	C7
Pugliese *et al.*[a]	298 K	105	75	/	75	/	84	60	71	/	58
Zheng *et al.* and this work	298 K[b]	99-106	82-85	71-73	84-87	75-77	75-76	73-77	67-72	61-62	57-62
	107 K[c]	98-107	82-86	73-75	80-83	74-76	76-77	76-78	67-71	60-63	56-61

^{13}C environment	Temperature	C8	C9	C10	C11	C12	C13	C14	C15	C16
Pugliese *et al.*[a]	298 K	71	60	17	171	21	173	29	29	173
Zheng *et al.* and this work	298 K[b]	68-71	73-74	17	169-171	21-22	172-173	29-30	31-32	175
	107 K[c]	70-72	73-75	15-17	168-170	18-22	170-173	26-30	30-32	173-176

Data acquired at [a] 9.4 T, 12.5 kHz MAS and 298 K from ref [2], [b] 9.4 T, 14 kHz MAS and 298 K from ref [3], and [c] 9.4 T, 8 kHz MAS and 107 K in this work.

Table S3: ^{1}H chemical shifts of HPMC-AS obtained by identifying the one bond ^{1}H-^{13}C correlations in 2D ^{1}H-^{13}C HETCOR spectra of HPMC-AS in the literature with the definitive ^{13}C spectral assignments of HPMC-AS obtained here.[4]

^{1}H environment	Chemical Shift (ppm)
H1	4.8
H2ether,3ether	2.5
H2ester,3ester,4,5,6ether,9	3.4
H6ester,7	3.0
H8	3.9
H10,12	1.1
H14,15	2.0

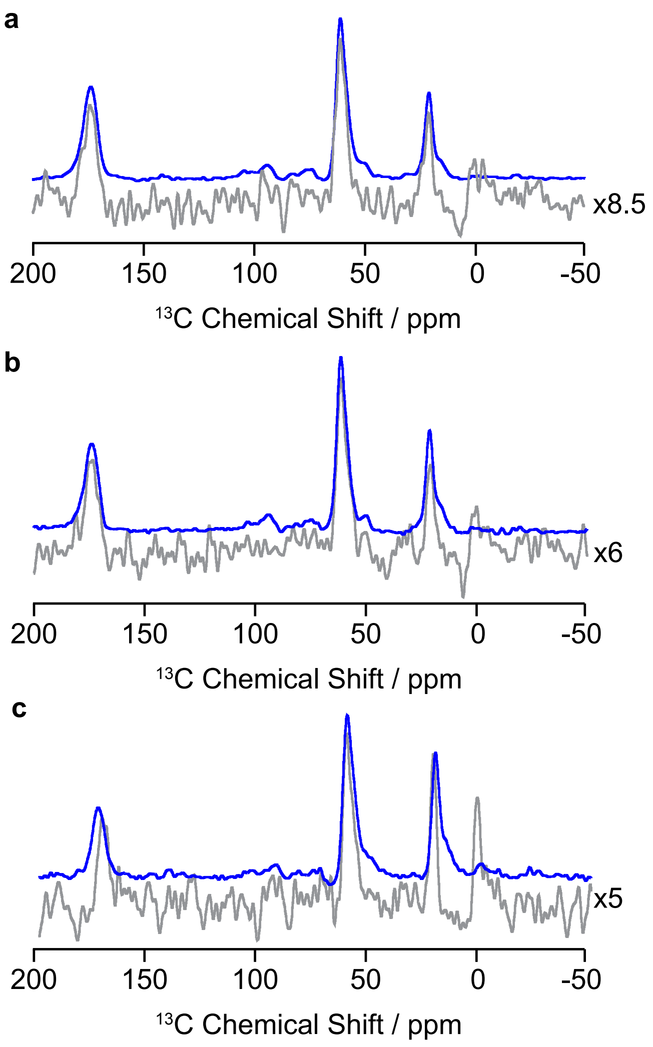

Figure S4: ^{13}C CP spectra with a dipolar dephasing echo filter for selective detection of quaternary and $^{13}CH_3$ signals for **a** HPMC-AS (LF), **b** HPMC-AS (MF) and **c** HPMC-AS (HF). The μw off (grey) spectra are scaled for clear comparisons with the μw on (blue) spectra. Signal at 1.8 ppm arises from the silicon plug used to seal the rotor. Spinning sidebands are indicated with asterisks (*).

The errors in enhancement are obtained using:

$$\Delta\varepsilon = \varepsilon\sqrt{\left(\frac{\Delta I_{\mu w\ ON}}{I_{\mu w\ ON}}\right)^2 + \left(\frac{\Delta I_{\mu w\ OFF}}{I_{\mu w\ OFF}}\right)^2}$$

where $\Delta I_{\mu w\ ON}$ and $\Delta I_{\mu w\ OFF}$ are the amplitude of the noise and $\Delta I_{\mu w\ ON}$ and $\Delta I_{\mu w\ OFF}$ are the signal amplitude with (μw on) and without (μw on) microwave irradiation.

REFERENCES

(1) Brownbill, N. J.; Gajan, D.; Lesage, A.; Emsley, L.; Blanc, F. Oxygen-17 dynamic nuclear polarisation enhanced solid-state NMR spectroscopy at 18.8 T. *Chem. Commun.* **2017**, *53* (17), 2563-2566.

(2) Pugliese, A.; Hawarden, L. E.; Abraham, A.; Tobyn, M.; Blanc, F. Solid state nuclear magnetic resonance studies of hydroxypropylmethylcellulose acetyl succinate polymer, a useful carrier in pharmaceutical solid dispersions. *Magn. Reson. Chem.* **2020**, *58* (11), 1036-1048.

(3) Zheng, Z.; Su, Y.; Schmidt-Rohr, K. Corrected solid-state ^{13}C nuclear magnetic resonance peak assignment and side-group quantification of hydroxypropyl methylcellulose acetyl succinate pharmaceutical excipients. *Magn. Reson. Chem.* **2023**, *61*, 595-605.

(4) Pugliese, A.; Toresco, M.; McNamara, D.; Iuga, D.; Abraham, A.; Tobyn, M.; Hawarden, L. E.; Blanc, F. Drug–polymer interactions in acetaminophen/hydroxypropylmethylcellulose acetyl succinate amorphous solid dispersions revealed by multidimensional multinuclear solid-state NMR spectroscopy. *Mol. Pharmaceutics* **2021**, *18* (9), 3519-3531.
